# Correction: Spatial quality control bypasses cell-based limitations on proteostasis to promote prion curing

**DOI:** 10.7554/eLife.06494

**Published:** 2015-01-28

**Authors:** Courtney L Klaips, Megan L Hochstrasser, Christine R Langlois, Tricia R Serio

Klaips CL, Hochstrasser ML, Langlois CR, Serio TR. 2014. Spatial quality control bypasses cell-based limitations on proteostasis to promote prion curing. *eLife* **3**:e04288. doi: 10.7554/eLife.04288

Published 9 December 2014

In the published article, the second column in Figure 5—figure supplement 1B was incorrectly labelled as ‘Sis1GFP’. This should have been labelled ‘Ssa1GFP’.

The article has been corrected accordingly.

